# Supplementary material for: The transcriptional regulatory network modulating human trophoblast stem cells to extravillous trophoblast differentiation
Source: Nat Commun. 2024 Feb 12;15:1285. doi: 10.1038/s41467-024-45669-2 (PMC10861538; doi:10.1038/s41467-024-45669-2)

Supplementary information for

The transcriptional regulatory network modulating human trophoblast stem cells to extravillous trophoblast differentiation

Mijeong Kim[1], Yu Jin Jang[1], Muyoung Lee[1], Qingqing Guo[1], Albert J. Son[1], Nikita A Kakkad[1], Abigail B Roland[1], Bum-Kyu Lee[2], Jonghwan Kim[1,3,*]

[1]Department of Molecular Biosciences, The University of Texas at Austin, Austin, TX 78712, USA
[2]Department of Biomedical Sciences, Cancer Research Center, University at Albany, State University of New York, Rensselaer, NY 12144, USA
[3]Lead contact
[*]Correspondence: jonghwankim@mail.utexas.edu

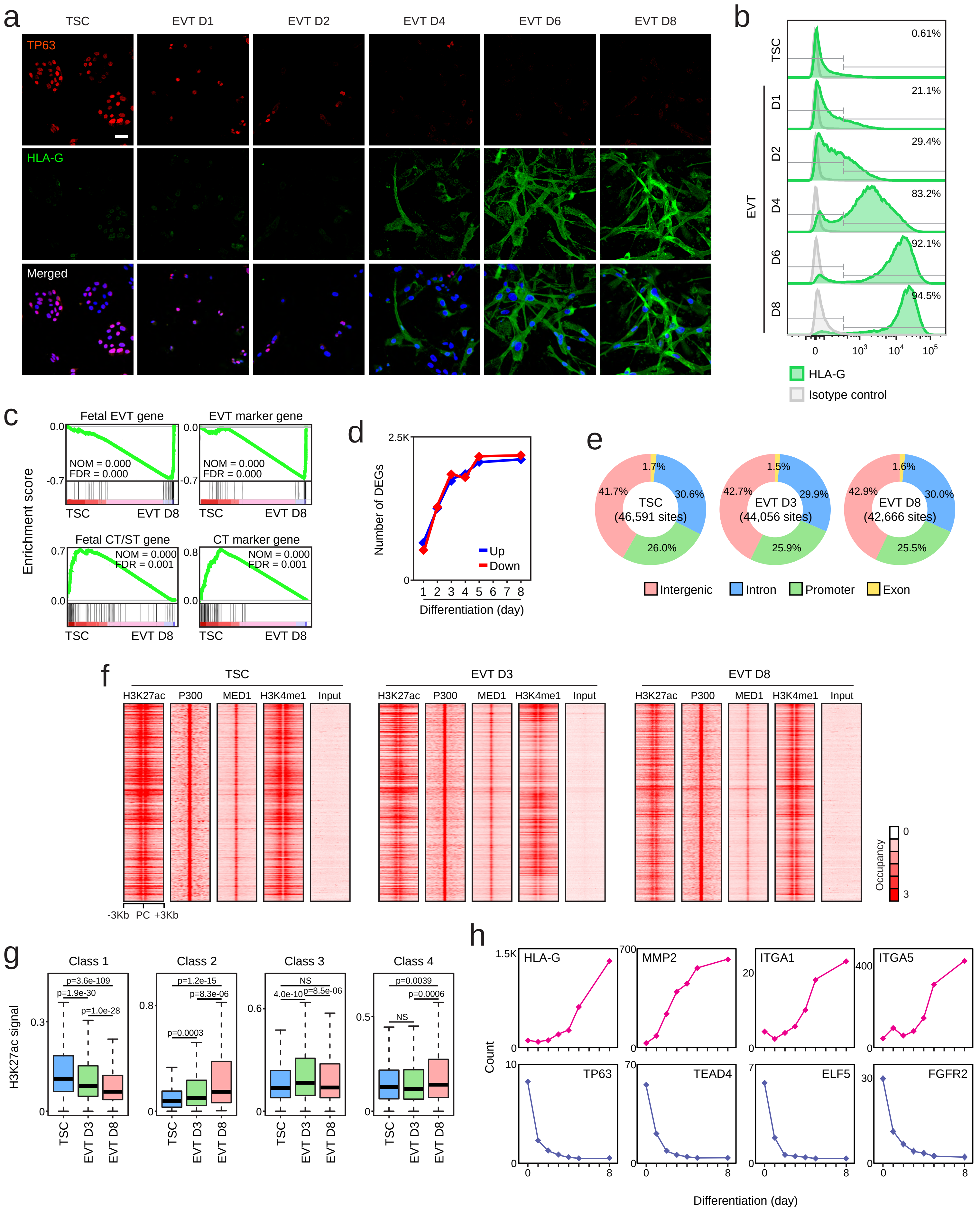
a
TSC
EVT D1
EVT D2
EVT D4
EVT D6
EVT D8
TP63
HLA-G
Merged
b
TSC
EVT
D1
D2
D4
D6
D8
0.61%
21.1%
29.4%
83.2%
92.1%
94.5%
HLA-G
Isotype control
c
Enrichment score
Fetal EVT gene
EVT marker gene
Fetal CT/ST gene
CT marker gene
NOM = 0.000
FDR = 0.000
FDR = 0.001
TSC
EVT D8
d
Number of DEGs
2.5K
Up
Down
Differentiation (day)
e
TSC (46,591 sites)
EVT D3 (44,056 sites)
EVT D8 (42,666 sites)
Intergenic
Intron
Promoter
Exon
f
H3K27ac
P300
MED1
H3K4me1
Input
-3Kb PC +3Kb
Occupancy
g
H3K27ac signal
Class 1
Class 2
Class 3
Class 4
p=3.6e-109
p=1.9e-30
p=1.0e-28
p=1.2e-15
p=8.3e-06
p=0.0003
NS
4.0e-10
p=8.5e-06
p=0.0039
p=0.0006
TSC
EVT D3
EVT D8
h
Count
HLA-G
MMP2
ITGA1
ITGA5
TP63
TEAD4
ELF5
FGFR2
Differentiation (day)

Supplementary Fig. 1 Dynamic changes in transcriptome and enhancer landscape during EVT differentiation.

a, Immunofluorescence of TP63 and HLA-G in TSCs and EVT differentiating cells on days 1 (EVT D1), 2 (EVT D2), 4 (EVT D4), 6 (EVT D6), and 8 (EVT D8). Scale bar: 100 μm. b, Flow cytometry analysis of surface HLA-G expression in TSCs and EVT differentiating cells. HLA-G expression was measured using a PE-conjugated HLA-G antibody and compared with the PE-conjugated isotype control. c, GSEA using Descartes fetal placenta extravillous trophoblasts (Fetal EVT) and Descartes fetal placenta syncytiotrophoblasts and villous cytotrophoblasts (Fetal CT/ST) gene sets, along with EVT and CT marker genes. The transcriptomes of EVT D8 and TSCs were compared to confirm the efficiency of EVT differentiation. NOM and FDR indicate nominal p-value and false discovery rate, respectively. d, Number of DEGs (with absolute $\log_2$-fold change > 1 and $p < 0.05$, compared to TSCs) in EVT differentiating cells. e, Genomic distribution of H3K27ac signals in TSCs, EVT D3, and EVT D8. f, Heatmap displaying enhancer signals revealed by ChIP-seq analysis of H3K27ac, P300, MED1, and H3K4me1 in TSCs, EVT D3, and EVT D8. The ChIP-seq signals of P300 were sorted first, and the peaks from other ChIP-seq experiments were plotted to the P300 binding loci. g, H3K27ac signals on class 1-4 genes in TSCs, EVT D3, and EVT D8. Significance by two-tailed Wilcoxon rank-sum test (NS, not significant). h, Expression changes of EVT marker genes (HLA-G, MMP2, ITGA1, and ITGA5) and TSC marker genes (TP63, TEAD4, ELF5, and EGFR2) during EVT differentiation measured by RNA-seq.

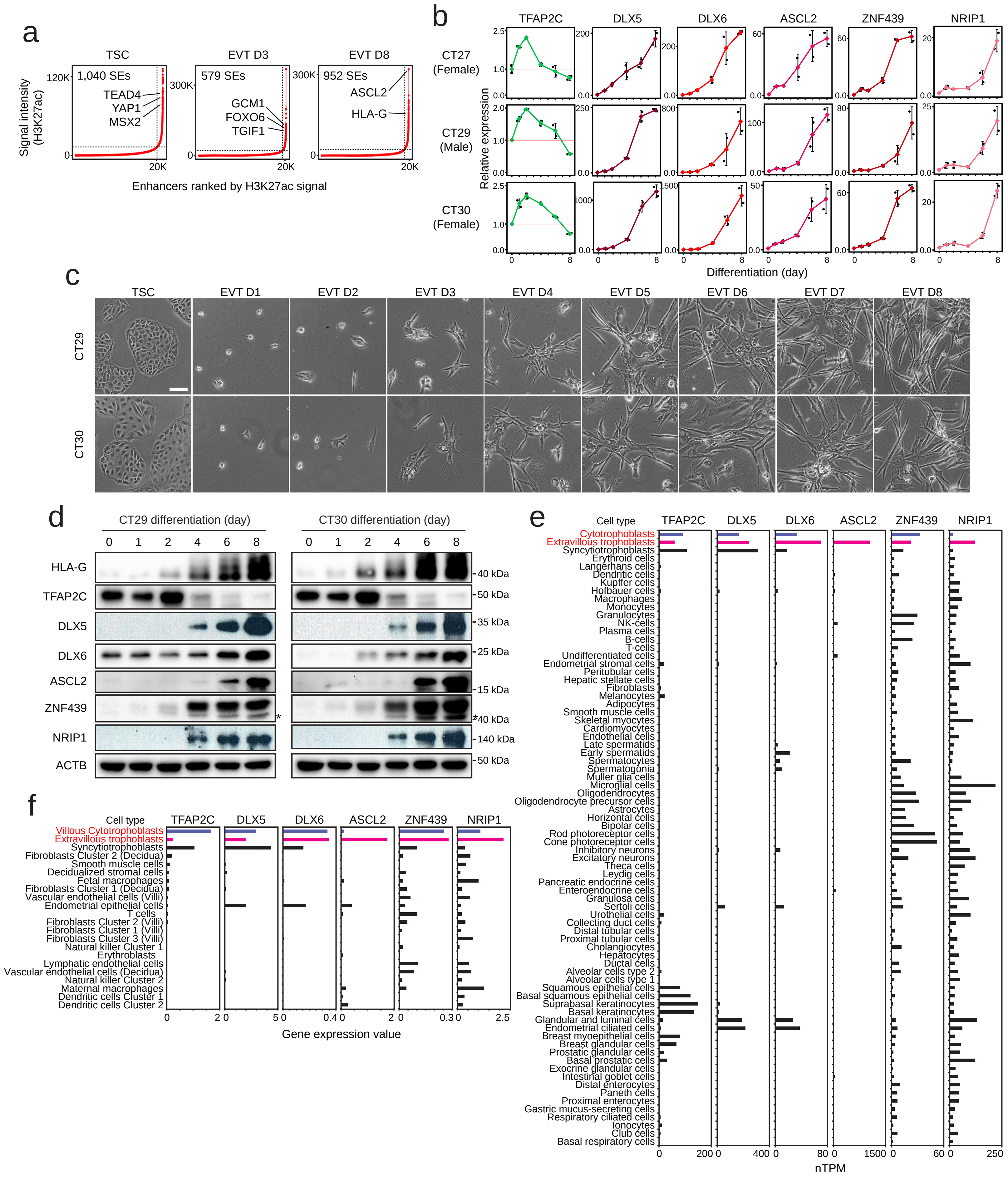
a
TSC
EVT D3
EVT D8
1,040 SEs
579 SEs
952 SEs
TEAD4
YAP1
MSX2
GCM1
FOXO6
TGIF1
ASCL2
HLA-G
120K
300K
300K
0
20K
Signal intensity (H3K27ac)
Enhancers ranked by H3K27ac signal
b
TFAP2C
DLX5
DLX6
ASCL2
ZNF439
NRIP1
CT27 (Female)
CT29 (Male)
CT30 (Female)
Relative expression
Differentiation (day)
c
TSC
EVT D1
EVT D2
EVT D3
EVT D4
EVT D5
EVT D6
EVT D7
EVT D8
CT29
CT30
d
CT29 differentiation (day)
CT30 differentiation (day)
0 1 2 4 6 8
HLA-G
TFAP2C
DLX5
DLX6
ASCL2
ZNF439
NRIP1
ACTB
40 kDa
50 kDa
35 kDa
25 kDa
15 kDa
40 kDa
140 kDa
50 kDa
e
Cell type
TFAP2C
DLX5
DLX6
ASCL2
ZNF439
NRIP1
Cytotrophoblasts
Extravillous trophoblasts
Syncytiotrophoblasts
Erythroid cells
Langerhans cells
Dendritic cells
Kupffer cells
Hofbauer cells
Macrophages
Monocytes
Granulocytes
NK-cells
Plasma cells
B-cells
T-cells
Undifferentiated cells
Endometrial stromal cells
Peritubular cells
Hepatic stellate cells
Fibroblasts
Melanocytes
Adipocytes
Smooth muscle cells
Skeletal myocytes
Cardiomyocytes
Endothelial cells
Late spermatids
Early spermatids
Spermatocytes
Spermatogonia
Muller glia cells
Microglial cells
Oligodendrocytes
Oligodendrocyte precursor cells
Astrocytes
Horizontal cells
Bipolar cells
Rod photoreceptor cells
Cone photoreceptor cells
Inhibitory neurons
Excitatory neurons
Theca cells
Leydig cells
Pancreatic endocrine cells
Enteroendocrine cells
Granulosa cells
Sertoli cells
Urothelial cells
Collecting duct cells
Distal tubular cells
Proximal tubular cells
Cholangiocytes
Hepatocytes
Ductal cells
Alveolar cells type 2
Alveolar cells type 1
Squamous epithelial cells
Basal squamous epithelial cells
Suprabasal keratinocytes
Basal keratinocytes
Glandular and luminal cells
Endometrial ciliated cells
Breast myoepithelial cells
Breast glandular cells
Prostatic glandular cells
Basal prostatic cells
Exocrine glandular cells
Intestinal goblet cells
Distal enterocytes
Paneth cells
Proximal enterocytes
Gastric mucus-secreting cells
Respiratory ciliated cells
Ionocytes
Club cells
Basal respiratory cells
nTPM
f
Cell type
TFAP2C
DLX5
DLX6
ASCL2
ZNF439
NRIP1
Villous Cytotrophoblasts
Extravillous trophoblasts
Syncytiotrophoblasts
Fibroblasts Cluster 2 (Decidua)
Smooth muscle cells
Decidualized stromal cells
Fetal macrophages
Fibroblasts Cluster 1 (Decidua)
Vascular endothelial cells (Villi)
Endometrial epithelial cells
T cells
Fibroblasts Cluster 2 (Villi)
Fibroblasts Cluster 1 (Villi)
Fibroblasts Cluster 3 (Villi)
Natural killer Cluster 1
Erythroblasts
Lymphatic endothelial cells
Vascular endothelial cells (Decidua)
Natural killer Cluster 2
Maternal macrophages
Dendritic cells Cluster 1
Dendritic cells Cluster 2
Gene expression value

Supplementary Fig. 2 Two classes of EVT regulators identified based on their expression and enhancer association.

a, Line graph presenting SE numbers based on ranked H3K27ac signals in TSCs and differentiating cells to EVT on days 3 (EVT D3) and 8 (EVT D8). b, Relative mRNA expression (fold change) of TFAP2C and late-stage TFs during EVT differentiation in CT-derived TSC lines. The fold change was calculated relative to the expression in TSCs for each individual cell line. Error bars: mean ± SD (n = 2, independent repeats). c, Representative brightfield images of TSCs and differentiating cells to EVT on days 1 to 8 in CT29 and CT30 TSC lines. The images were captured in two independent experiments, both demonstrating consistent results. Scale bar: 100 μm. d, Western blot analysis showing the expression patterns of EVT regulator candidates in CT29 and CT30 lines. HLA-G expression was measured to validate EVT differentiation. ACTB was used as a loading control. *ZNF439 (lower band). Independent repeats, utilizing CT27 TSC line, were conducted as presented in Fig. 2e. e and f, Normalized transcripts per million values (nTPM) (e) and gene expression value (f) of EVT regulator candidates in individual cell types from single-cell type transcriptomic map of human tissues, retrieved from the Human Protein Atlas and PlacentaCellEnrich tool.

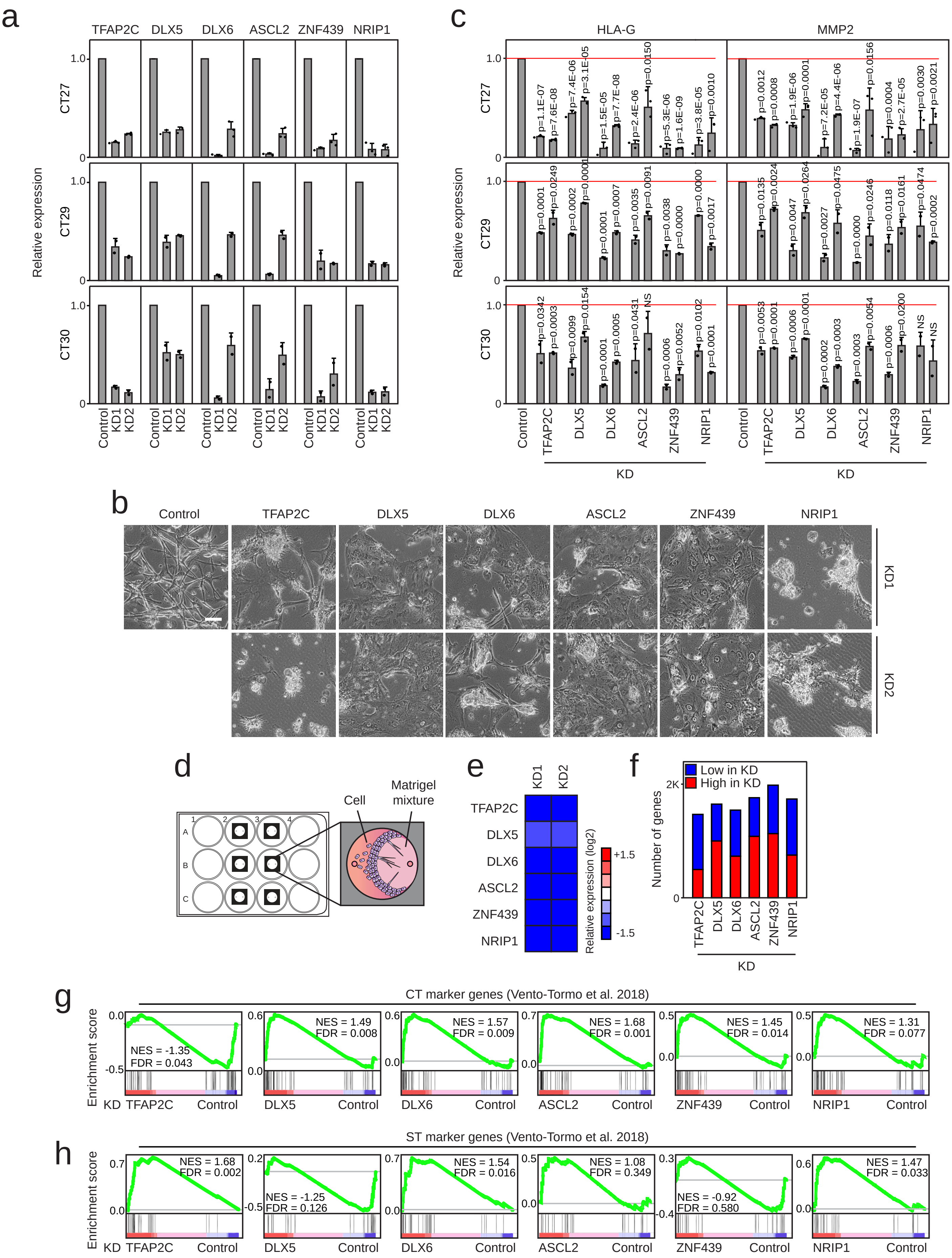

a
TFAP2C DLX5 DLX6 ASCL2 ZNF439 NRIP1
CT27
CT29
CT30
Relative expression
Control KD1 KD2
c
HLA-G
MMP2
Relative expression
CT27
CT29
CT30
Control TFAP2C DLX5 DLX6 ASCL2 ZNF439 NRIP1
KD
b
Control TFAP2C DLX5 DLX6 ASCL2 ZNF439 NRIP1
KD1
KD2
d
Cell
Matrigel mixture
e
KD1 KD2
TFAP2C DLX5 DLX6 ASCL2 ZNF439 NRIP1
Relative expression (log2)
+1.5
-1.5
f
Low in KD
High in KD
Number of genes
2K
0
TFAP2C DLX5 DLX6 ASCL2 ZNF439 NRIP1
KD
g
CT marker genes (Vento-Tormo et al. 2018)
Enrichment score
NES = -1.35 FDR = 0.043
NES = 1.49 FDR = 0.008
NES = 1.57 FDR = 0.009
NES = 1.68 FDR = 0.001
NES = 1.45 FDR = 0.014
NES = 1.31 FDR = 0.077
KD TFAP2C Control
DLX5 Control
DLX6 Control
ASCL2 Control
ZNF439 Control
NRIP1 Control
h
ST marker genes (Vento-Tormo et al. 2018)
Enrichment score
NES = 1.68 FDR = 0.002
NES = -1.25 FDR = 0.126
NES = 1.54 FDR = 0.016
NES = 1.08 FDR = 0.349
NES = -0.92 FDR = 0.580
NES = 1.47 FDR = 0.033
KD TFAP2C Control
DLX5 Control
DLX6 Control
ASCL2 Control
ZNF439 Control
NRIP1 Control

Supplementary Fig. 3 Requirement of both early and late-stage TFs for EVT differentiation.
a, Relative mRNA expression showing KD efficiencies of target TFs in CT27, CT29, and CT30 TSC lines, compared to the respective controls. Error bars: mean ± SD (n = 3 independent repeats for the CT27 line and n = 2 independent repeats for CT29 and CT30 lines). b, Representative brightfield images of EVTs after KD of individual candidates of EVT regulator and the control. The images were captured in two independent experiments, both demonstrating consistent results. Scale bar: 100 μm. c, Relative mRNA expression of EVT marker genes in EVTs after KD of individual candidates of EVT regulator, differentiated from CT27, CT29, or CT30 TSC lines. Error bars: mean ± SD (n = 3 independent repeats for the CT27 line and n = 2 independent repeats for CT29 and CT30 lines). Significance by two-sided Student's t-test (NS, not significant). d, Schematic of the invasion assay chamber system. e, Heatmap displaying KD efficiencies of target TFs in the RNA-seq data. f, Number of genes with significantly lower (blue) or higher (red) expression in individual KD EVTs compared to the controls. g and h, GSEA utilizing (g) CT and (h) ST marker genes defined in scRNA-seq data of the human first-trimester placenta to compare EVT factor KD cells with the control. NES, normalized enrichment score. FDR, false discovery rate.

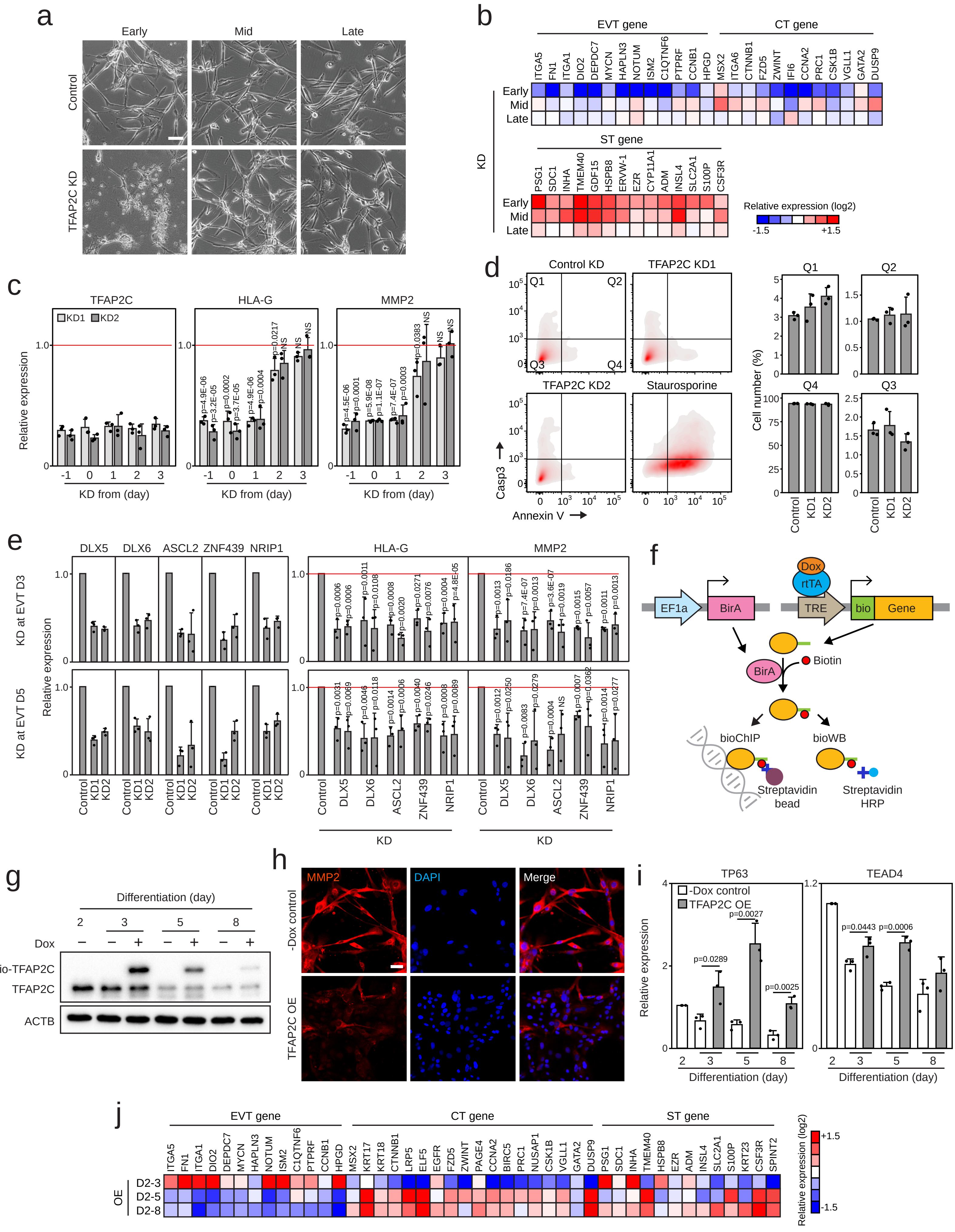

a
Early
Mid
Late
Control
TFAP2C KD
b
EVT gene
CT gene
ITGA5
FN1
ITGA1
DIO2
DEPDC7
MYCN
HAPLN3
NOTUM
ISM2
C1QTNF6
PTPRF
CCNB1
HPGD
MSX2
ITGA6
CTNNB1
FZD5
ZWINT
IFI6
CCNA2
PRC1
CSK1B
VGLL1
GATA2
DUSP9
Early
Mid
Late
KD
ST gene
PSG1
SDC1
INHA
TMEM40
GDF15
HSPB8
ERVW-1
EZR
CYP11A1
ADM
INSL4
SLC2A1
S100P
CSF3R
Relative expression (log2)
-1.5
+1.5
c
TFAP2C
HLA-G
MMP2
KD1
KD2
Relative expression
KD from (day)
d
Control KD
TFAP2C KD1
TFAP2C KD2
Staurosporine
Q1
Q2
Q3
Q4
Casp3
Annexin V
Cell number (%)
Control
KD1
KD2
e
DLX5
DLX6
ASCL2
ZNF439
NRIP1
HLA-G
MMP2
KD at EVT D3
KD at EVT D5
Relative expression
Control
KD
f
EF1a
BirA
Dox
rtTA
TRE
bio
Gene
Biotin
bioChIP
bioWB
Streptavidin bead
Streptavidin HRP
g
Differentiation (day)
Dox
bio-TFAP2C
TFAP2C
ACTB
h
MMP2
DAPI
Merge
-Dox control
TFAP2C OE
i
TP63
TEAD4
-Dox control
TFAP2C OE
Relative expression
Differentiation (day)
j
EVT gene
CT gene
ST gene
ITGA5
FN1
ITGA1
DIO2
DEPDC7
MYCN
HAPLN3
NOTUM
ISM2
C1QTNF6
PTPRF
CCNB1
HPGD
MSX2
KRT17
KRT18
CTNNB1
LRP5
ELF5
EGFR
FZD5
ZWINT
PAGE4
CCNA2
BIRC5
PRC1
NUSAP1
CSK1B
VGLL1
GATA2
DUSP9
PSG1
SDC1
INHA
TMEM40
HSPB8
EZR
ADM
INSL4
SLC2A1
S100P
KRT23
CSF3R
SPINT2
OE
D2-3
D2-5
D2-8
Relative expression (log2)
+1.5
-1.5

Supplementary Fig. 4 Stage-specific regulation of TFAP2C during EVT differentiation.
a, Representative brightfield images of EVTs after TFAP2C KD at different time points and individual controls. Early: KD day -1-8, Mid: KD day 3-8, Late: KD day 5-8. The images were captured in two independent experiments, both demonstrating consistent results. Scale bar: 100 μm. b, Heatmaps of relative expression of EVT, CT, and ST marker genes in EVT day 8 cells infected with TFAP2C-targeting lentivirus at different time points: day -1 (KD-Early), day 3 (KD-Mid), and day 5 (KD-Late), relative to control. c, Relative mRNA expression of TFAP2C and EVT marker genes in EVTs infected with TFAP2C-targeting lentivirus at various time points (days 1, 0, 1, 2, and 3) during EVT differentiation, compared to control. Error bars: mean ± SD (n = 3, independent repeats). Significance by Student's t-test (NS, not significant). d, Flow cytometry density plots of TSCs displaying Annexin V and caspase-3 activity after TFAP2C-targeting lentivirus infection, with staurosporine (1 μM, a potent inducer of apoptosis) as a control. Error bars: mean ± SD (n = 3, independent repeats). e, KD efficiency of late-stage TFs and expression levels of EVT marker genes in EVTs infected with lentiviral targeting individual late-stage TFs on days 3 and 5, compared to control. Error bars: mean ± SD (n = 3, independent repeats). Significance by Student's t-test (NS, not significant). f, Schematic of the pSBFB system for bioChIP and bioWB. g, Western blot analysis of endogenous and exogenous (bio-TFAP2C) TFAP2C expression at different time points. Due to the biotinylation sequence (~6 kDa), bio-TFAP2C is larger than the endogenous protein. Dox was treated on day 2, and TFAP2C expression was detected in cells on days 3, 5, and 8 of differentiation. h, Immunofluorescence of MMP2 in TFAP2C overexpressing EVTs (day 2-8) and -Dox control. Scale bar: 100 μm. i, Relative mRNA expression of TSC marker genes in TFAP2C OE and individual controls. Dox was treated on day 2, and the expression was detected on days 3, 5, and 8 of differentiation. Error bars: mean ± SD (n = 3, independent repeats). Significance by Student's t-test. j, Heatmap of relative expression of EVT, CT, and ST marker genes in TFAP2C OE cells compared to individual -Dox control. Dox (1 μg/mL) was treated on day 2 of EVT differentiation, and cells were collected on days 3 (D2-3), 5 (D2-5), and 8 (D2-8).

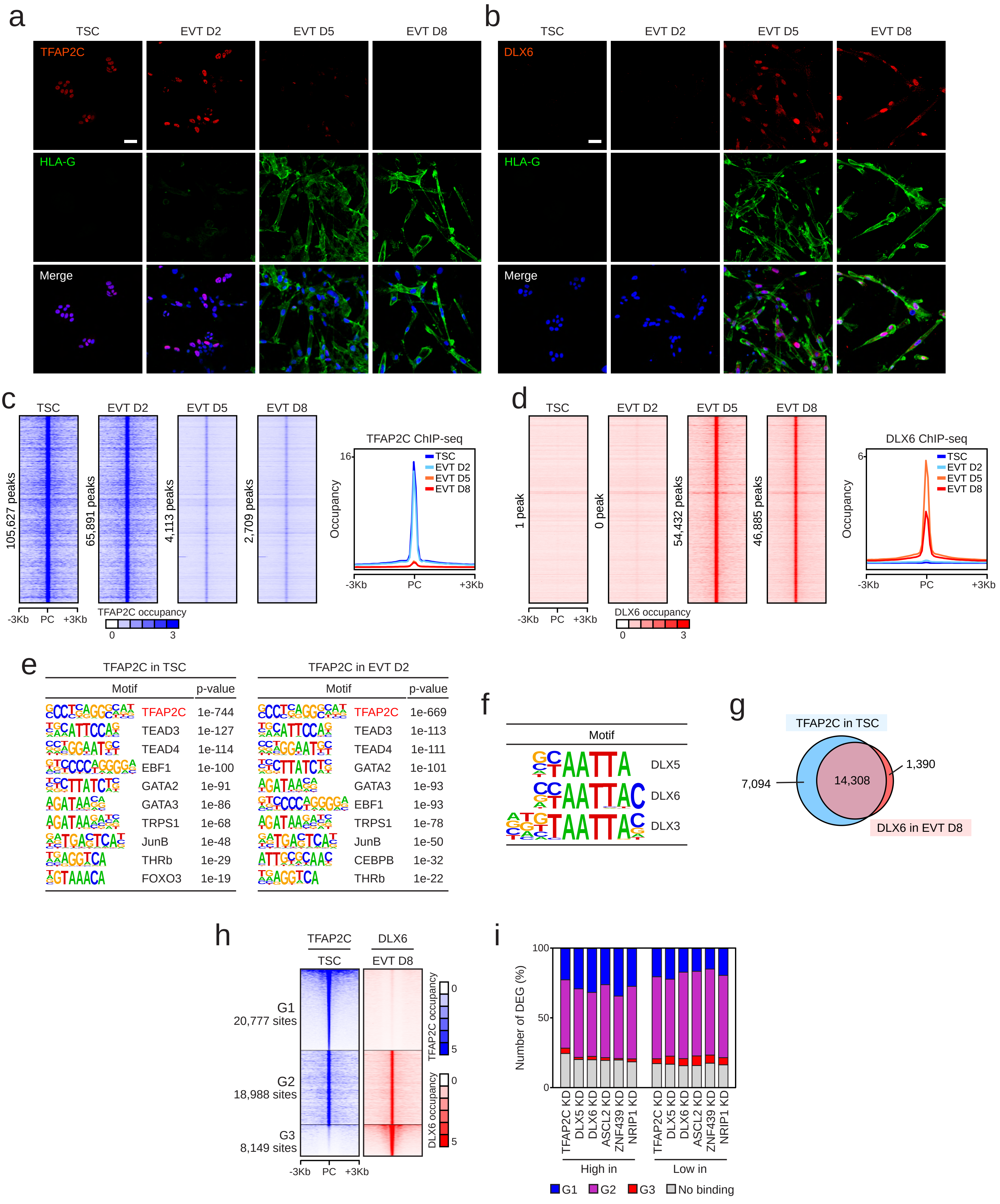
a
TSC
EVT D2
EVT D5
EVT D8
TFAP2C
HLA-G
Merge
b
TSC
EVT D2
EVT D5
EVT D8
DLX6
HLA-G
Merge
c
TSC
EVT D2
EVT D5
EVT D8
105,627 peaks
65,891 peaks
4,113 peaks
2,709 peaks
-3Kb PC +3Kb
TFAP2C occupancy
0 3
TFAP2C ChIP-seq
16
Occupancy
TSC
EVT D2
EVT D5
EVT D8
-3Kb PC +3Kb
d
TSC
EVT D2
EVT D5
EVT D8
1 peak
0 peak
54,432 peaks
46,885 peaks
-3Kb PC +3Kb
DLX6 occupancy
0 3
DLX6 ChIP-seq
6
Occupancy
TSC
EVT D2
EVT D5
EVT D8
-3Kb PC +3Kb
e
TFAP2C in TSC
Motif p-value
TFAP2C 1e-744
TEAD3 1e-127
TEAD4 1e-114
EBF1 1e-100
GATA2 1e-91
GATA3 1e-86
TRPS1 1e-68
JunB 1e-48
THRb 1e-29
FOXO3 1e-19
TFAP2C in EVT D2
Motif p-value
TFAP2C 1e-669
TEAD3 1e-113
TEAD4 1e-111
GATA2 1e-101
GATA3 1e-93
EBF1 1e-93
TRPS1 1e-78
JunB 1e-50
CEBPB 1e-32
THRb 1e-22
f
Motif
DLX5
DLX6
DLX3
g
TFAP2C in TSC
7,094
14,308
1,390
DLX6 in EVT D8
h
TFAP2C
TSC
DLX6
EVT D8
G1
20,777 sites
G2
18,988 sites
G3
8,149 sites
-3Kb PC +3Kb
TFAP2C occupancy
0 5
DLX6 occupancy
0 5
i
Number of DEG (%)
100
50
0
TFAP2C KD
DLX5 KD
DLX6 KD
ASCL2 KD
ZNF439 KD
NRIP1 KD
High in
Low in
G1 G2 G3 No binding

Supplementary Fig. 5 TFAP2C primes the late-stage TFs and EVT-active genes at the early-stage of differentiation.

a and b, Immunofluorescence of (a) TFAP2C and (b) DLX6 in TSCs and differentiating cells to EVT on days 2 (EVT D2), 5 (EVT D5), and 8 (EVT D8). c and d, Heatmaps illustrating the occupancy signals of (c) TFAP2C and (d) DLX6 in TSCs, EVT D1, EVT D2, EVT D5, and EVT D8. e, Enriched motifs in TFAP2C ChIP-seq peaks in TSCs (left) and EVT D2 (right). Motifs identified using the findMotifsGenome.pl module under HOMER (v4.11). f, DLX3, DLX5, and DLX6 motifs. g, Venn diagram illustrating distinct and shared target genes of TFAP2C in TSCs and DLX6 in EVTs. h, Heatmaps showing TFAP2C unique target loci in TSCs (group 1, G1), TFAP2C/DLX6 common target loci (group 2, G2), and DLX6 unique target loci in EVT D8(group 3, G3). PC, peak center. i, Bar graphs displaying the number of DEGs in EVTs following KD of individual EVT TFs directly bound by TFAP2C in TSCs (group 1, G1 targets), common to both TFAP2C and DLX6 (group 2, G2 targets), or specifically bound by DLX6 in EVT D8 (group 3, G3 targets), allowing identification of DEGs directly regulated by TFAP2C and DLX6.

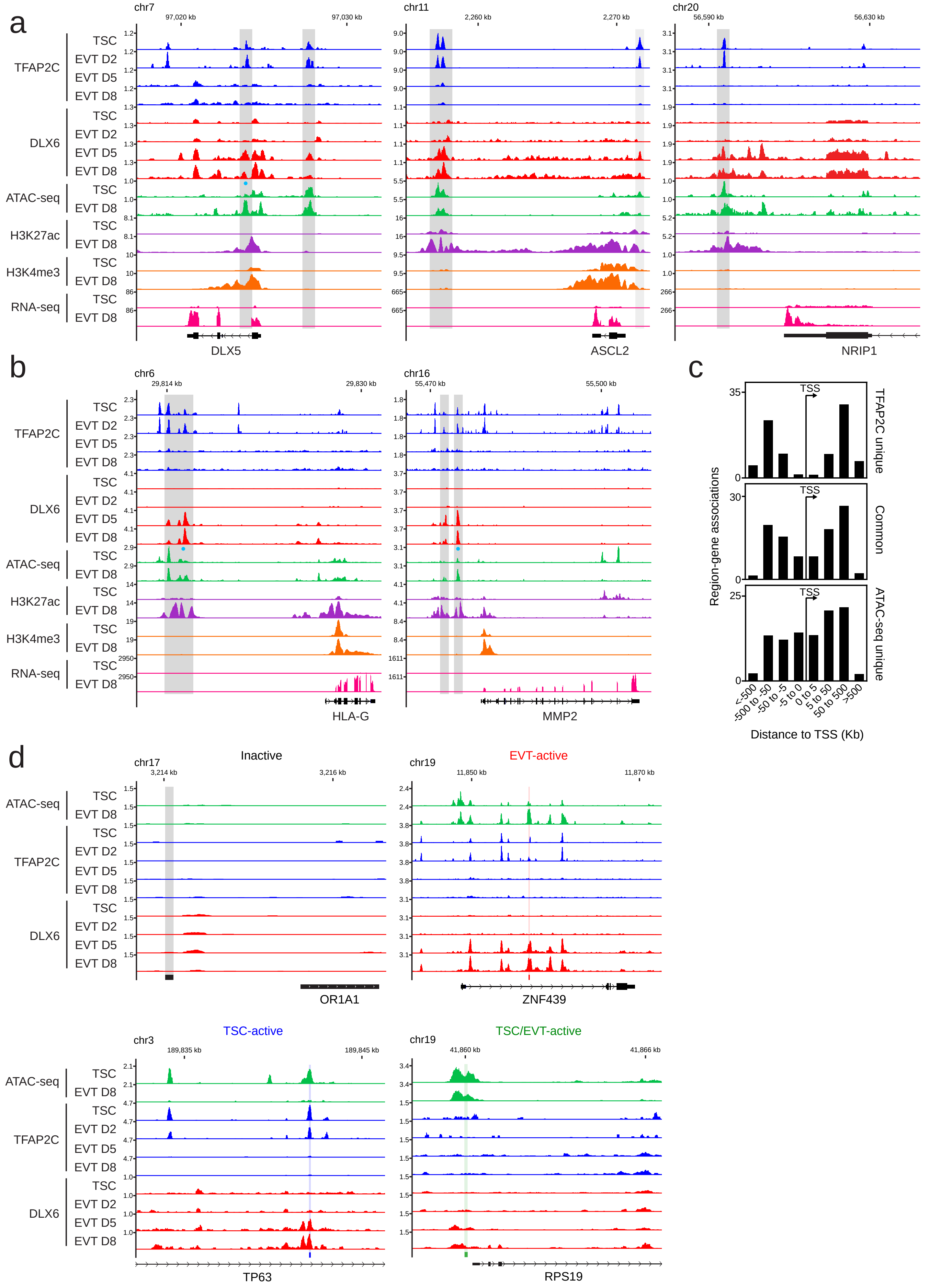

a
TFAP2C
DLX6
ATAC-seq
H3K27ac
H3K4me3
RNA-seq
TSC
EVT D2
EVT D5
EVT D8
chr7
97,020 kb
97,030 kb
DLX5
chr11
2,260 kb
2,270 kb
ASCL2
chr20
56,590 kb
56,630 kb
NRIP1
b
chr6
29,814 kb
29,830 kb
HLA-G
chr16
55,470 kb
55,500 kb
MMP2
c
Region-gene associations
TSS
TFAP2C unique
Common
ATAC-seq unique
<-500
-500 to -50
-50 to -5
-5 to 0
0 to 5
5 to 50
50 to 500
>500
Distance to TSS (Kb)
d
Inactive
chr17
3,214 kb
3,216 kb
OR1A1
EVT-active
chr19
11,850 kb
11,870 kb
ZNF439
TSC-active
chr3
189,835 kb
189,845 kb
TP63
TSC/EVT-active
chr19
41,860 kb
41,866 kb
RPS19

Supplementary Fig. 6 Pioneering activities of TFAP2C on the late-stage TFs and EVT-active genes. a and b Gene track view of TFAP2C (blue) and DLX6 (red) signals in TSCs and differentiating cells to EVT on days 2 (EVT D2), 5 (EVT D5), and 8 (EVT D8), along with ATAC-seq (green), H3K27ac (violet), H3K4me3 (orange) signals in TSCs and EVT D8. mRNA expression levels (pink) in TSCs and EVT D8 are displayed at the loci near (a) EVT factors and (b) EVT marker genes. c, Distance to the transcription start site (TSS) of top 3,000 TFAP2C ChIP-seq unique, common, and ATAC-seq unique peaks in TSCs. d, Gene track view illustrating ATAC-seq signals (green) in TSCs and EVT D8, along with TFAP2C (blue) and DLX6 (red) ChIP-seq signals in TSCs, EVT D2, EVT D5, and EVT D8. ATAC-qPCR targets were labeled for inactive (black), EVT-active (red), TSC-active (blue), and TSC/EVT-active (green) loci.

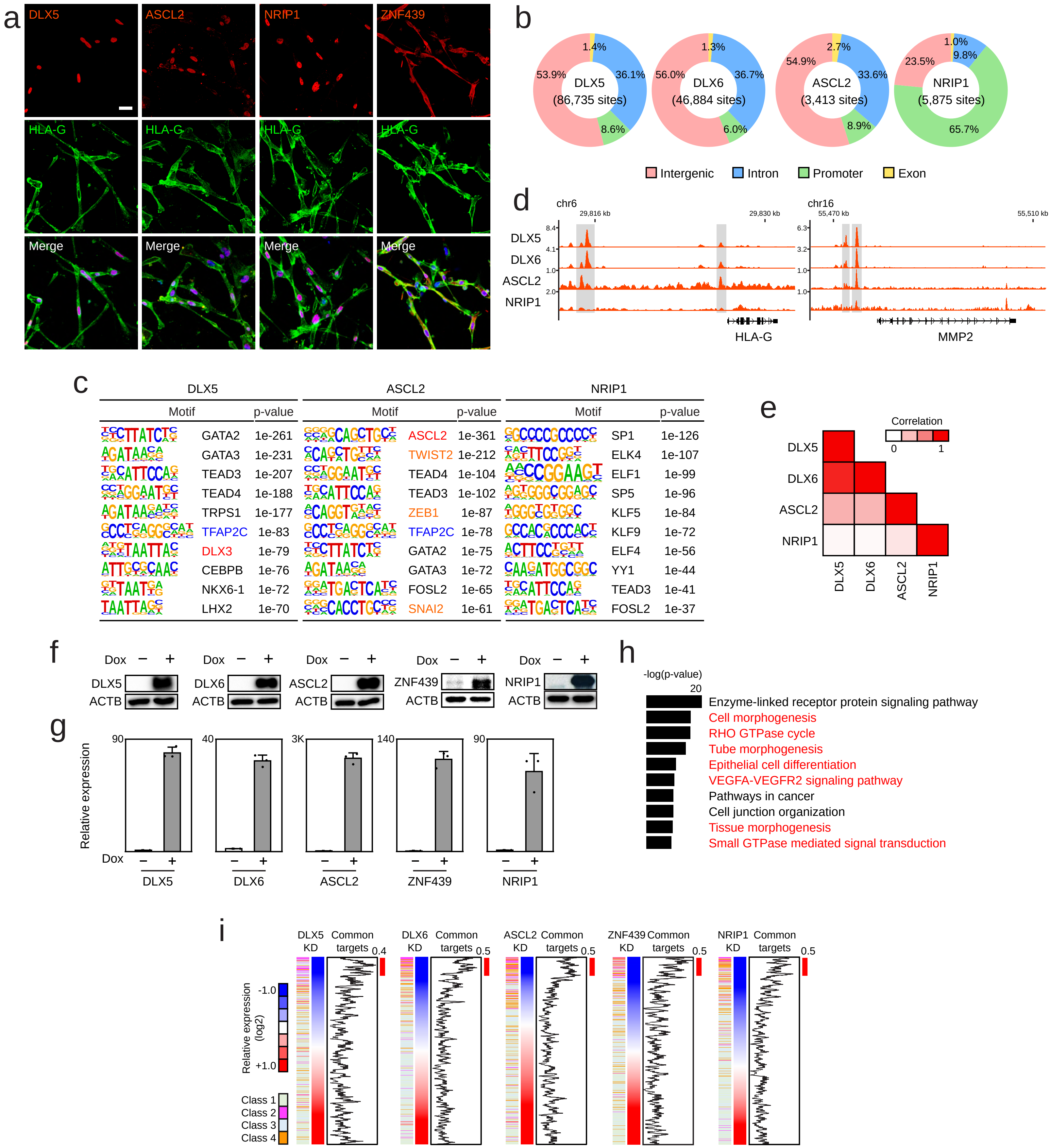
a
DLX5
ASCL2
NRIP1
ZNF439
HLA-G
Merge
b
DLX5 (86,735 sites)
53.9%
36.1%
1.4%
8.6%
DLX6 (46,884 sites)
56.0%
36.7%
1.3%
6.0%
ASCL2 (3,413 sites)
54.9%
33.6%
2.7%
8.9%
NRIP1 (5,875 sites)
23.5%
9.8%
1.0%
65.7%
Intergenic
Intron
Promoter
Exon
d
chr6
29,816 kb
29,830 kb
chr16
55,470 kb
55,510 kb
DLX5
DLX6
ASCL2
NRIP1
HLA-G
MMP2
c
DLX5
Motif p-value
GATA2 1e-261
GATA3 1e-231
TEAD3 1e-207
TEAD4 1e-188
TRPS1 1e-177
TFAP2C 1e-83
DLX3 1e-79
CEBPB 1e-76
NKX6-1 1e-72
LHX2 1e-70
ASCL2
Motif p-value
ASCL2 1e-361
TWIST2 1e-212
TEAD4 1e-104
TEAD3 1e-102
ZEB1 1e-87
TFAP2C 1e-78
GATA2 1e-75
GATA3 1e-72
FOSL2 1e-65
SNAI2 1e-61
NRIP1
Motif p-value
SP1 1e-126
ELK4 1e-107
ELF1 1e-99
SP5 1e-96
KLF5 1e-84
KLF9 1e-72
ELF4 1e-56
YY1 1e-44
TEAD3 1e-41
FOSL2 1e-37
e
Correlation
0
1
DLX5
DLX6
ASCL2
NRIP1
f
Dox − +
DLX5
DLX6
ASCL2
ZNF439
NRIP1
ACTB
g
Relative expression
90
40
3K
140
90
Dox − +
DLX5
DLX6
ASCL2
ZNF439
NRIP1
h
-log(p-value)
20
Enzyme-linked receptor protein signaling pathway
Cell morphogenesis
RHO GTPase cycle
Tube morphogenesis
Epithelial cell differentiation
VEGFA-VEGFR2 signaling pathway
Pathways in cancer
Cell junction organization
Tissue morphogenesis
Small GTPase mediated signal transduction
i
DLX5 KD
DLX6 KD
ASCL2 KD
ZNF439 KD
NRIP1 KD
Common targets
0.4
0.5
Relative expression (log2)
-1.0
+1.0
Class 1
Class 2
Class 3
Class 4

Supplementary Fig. 7 Late-stage TFs collaborate on regulating EVT-specific gene expression program.

a, Immunofluorescence of late-stage TFs and HLA-G in mature EVTs (EVT D8). b, Pie charts depicting the genomic distribution of the binding loci of late-stage TFs. c, Enriched motifs in the peaks of each late-stage TF in EVT D8 using the findMotifsGenome.pl module under HOMER (v4.11). d, Gene track view showing near the EVT marker genes (HLA-G and MMP2). e, Correlation heatmap for all pairwise comparisons of late-stage TF target genes. f, Western blot analysis using native antibodies detecting individual late-stage TFs to show induction of target proteins. g, Relative mRNA expression of late-stage TFs, showing induction levels in the cells overexpressing individual late-stage TFs in TSCs. The late-stage TFs were induced by administration of doxycycline (Dox) for 6 days in TSC culture condition. Error bars: mean ± SD (n = 3, independent repeats). h, GO analysis of the common targets of DLX5, DLX6, and ASCL2. i, Heatmaps representing expression of class 1-4 genes in the EVTs after KD of individual late-stage TFs. Genes were ordered according to relative gene expression levels in the KD cells, compared to control (from lowest to highest). The number of occupancies of DLX5, DLX6, and ASCL2 (value: 0-3) was plotted as a moving average (window size: 50). Red bars indicate enriched occupancies.

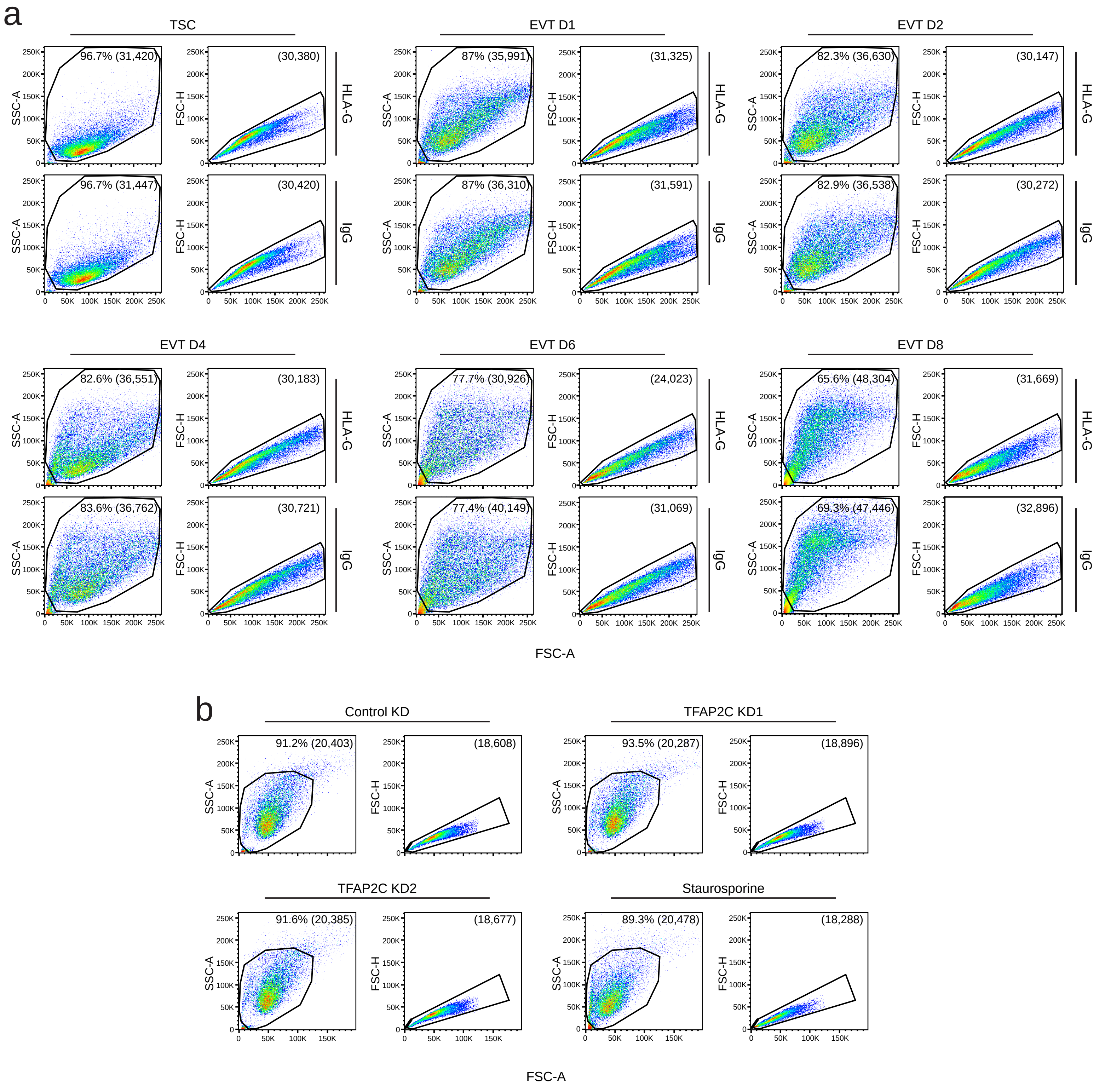
a
TSC
EVT D1
EVT D2
EVT D4
EVT D6
EVT D8
HLA-G
IgG
SSC-A
FSC-H
FSC-A
96.7% (31,420)
(30,380)
96.7% (31,447)
(30,420)
87% (35,991)
(31,325)
87% (36,310)
(31,591)
82.3% (36,630)
(30,147)
82.9% (36,538)
(30,272)
82.6% (36,551)
(30,183)
83.6% (36,762)
(30,721)
77.7% (30,926)
(24,023)
77.4% (40,149)
(31,069)
65.6% (48,304)
(31,669)
69.3% (47,446)
(32,896)
b
Control KD
TFAP2C KD1
TFAP2C KD2
Staurosporine
91.2% (20,403)
(18,608)
93.5% (20,287)
(18,896)
91.6% (20,385)
(18,677)
89.3% (20,478)
(18,288)
SSC-A
FSC-H
FSC-A

Supplementary Fig. 8 Gating strategy for assessing HLA-G and Annexin V/caspase-3 signals.
a and b, To exclude dead cells, all samples were initially gated using FSC-A/SSC-A. Single cells were then selected by gating FSC-A/FSC-H to remove cell doublets. a, HLA-G expression of TSCs and differentiating cells to EVTs were measured. Cells stained with IgG were used as the control. b, Signal intensity of Annexin V and caspase-3 activities in TSCs infected with lentivirus particles targeting TFAP2C were compared with control KD and Staurosporine-treated cells (1 μM).

Programs or code utilized in this study and associated figures

ChIP-seq, bioChIP-seq, and ATAC-seq data processing and analysis

- Alignment of sequencing data: Bowtie2 (v2.4.5)[1] with default settings.
- Filtering aligned reads: SAMtools[2] with a quality value threshold of > 10.
- Excluding peaks in simple redundant regions: peaks identified within the repeat-mask file for the human genome hg38 (https://genome.ucsc.edu/cgi-bin/hgTables) were removed.
- Generation of normalized wig file: deepTools[3] utilizing the bamCoverage tool with bin size 10, CPM, and extendReads 67 options.
- Visualization of peaks (Fig. 2b, 5d, 6a, 7a, Supplementary Fig. 6a, 6b, 6d, 7d): IGV program[4].
- Peak calling: MACS3[5] with default settings.
- Motif identification: HOMER (v4.11)[6] findMotifsGenome.pl module. For histone ChIP-seq data, a region size of 500 bp (Fig. 2d) and for ChIP-seq data, a region size of 200 bp (Fig. 5c and Supplementary Fig. 5e).
- Comparison of ChIP-seq and ATAC-seq peaks or ChIP-seq peaks: MAnorm program (v1.3.0)[7] with default settings using MACS3 peaks and bed files as inputs (Fig. 6b and Supplementary Fig. 5h).
- GO enrichment analysis of ChIP-seq peaks (Fig. 6c and Supplementary Fig. 7h): GREAT software[8] with the whole genome as background regions.
- Identification of super-enhancers (Supplementary Fig. 2a and Supplementary Data 3): ROSE program with default settings and -s 12500 and -t 2500 options using MACS3 peaks as input.
- Correlation among late-stage TF target genes (Supplementary Fig. 7e): R package Corrr (Kuhn, M., Jackson, S., & Cimentada, J. (2020). corrr: Correlations in R. R Package version 0.4, 2.) and pheatmap (v1.0.12, Kolde, R. (2019). pheatmap: Pretty Heatmaps. R package version 1.0. 12. CRAN. R-project. org/package= pheatmap.)

RNA-seq data processing and analysis

- Alignment of sequencing data: salmon (v1.4.0)[9] to human transcript (hg38).
- Calculation of TPM value: R package tximport using salmon count results as input and DESeq2 (v1.30.1)[10] using the median of the ratio method.
- Clustering analysis of time-course transcriptome analysis of EVT differentiating cells (Fig. 1d): Dirichlet Process Gaussian process (DPGP) mixture model[11]. Program and code: https://github.com/PrincetonUniversity/DP_GP_cluster.
- GO and pathway enrichment analysis (Fig. 3e and 3g): DAVID[12] and Metascape[13] (Fig. 1e).
- GSEA analysis (Fig. 3d, 4i and Supplementary Fig. 1c, 3g, 3h): GSEA program (https://www.gsea-msigdb.org/gsea/index.jsp) using CT, ST, and EVT marker gene sets defined in scRNA-seq data of the human first-trimester placenta[14,15].
- Sample-to-sample distance heatmap (Fig. 1f): R package DESeq2 (v1.30.1)[10].

Visualization of analysis results

- Visualization of heatmaps for Fig. 1d, 3c and Supplementary Fig. 3e, 4b, 4j, 7e: Java TreeView[16].

- Visualization of heatmaps for Fig. 5e, 5f, 5g, 5h, 6b and Supplementary Fig. 1f, 5c, 5d: HOMER (v4.11)[6] annotatePeaks.pl module with default settings and Java TreeView[16].

References

1. Langmead, B. & Salzberg, S.L. Fast gapped-read alignment with Bowtie 2. *Nat Methods* **9**, 357-359 (2012).
2. Li, H. et al. The Sequence Alignment/Map format and SAMtools. *Bioinformatics* (Oxford, England) **25**, 2078-2079 (2009).
3. Ramírez, F. et al. deepTools2: a next generation web server for deep-sequencing data analysis. *Nucleic Acids Res* **44**, W160-165 (2016).
4. Robinson, J.T. et al. Integrative genomics viewer. *Nat Biotechnol* **29**, 24-26 (2011).
5. Zhang, Y. et al. Model-based analysis of ChIP-Seq (MACS). *Genome Biol* **9**, R137 (2008).
6. Heinz, S. et al. Simple combinations of lineage-determining transcription factors prime cis-regulatory elements required for macrophage and B cell identities. *Mol Cell* **38**, 576-589 (2010).
7. Shao, Z., Zhang, Y., Yuan, G.C., Orkin, S.H. & Waxman, D.J. MAnorm: a robust model for quantitative comparison of ChIP-Seq data sets. *Genome Biol* **13**, R16 (2012).
8. McLean, C.Y. et al. GREAT improves functional interpretation of cis-regulatory regions. *Nat Biotechnol* **28**, 495-501 (2010).
9. Patro, R., Duggal, G., Love, M.I., Irizarry, R.A. & Kingsford, C. Salmon provides fast and bias-aware quantification of transcript expression. *Nat Methods* **14**, 417-419 (2017).
10. Love, M.I., Huber, W. & Anders, S. Moderated estimation of fold change and dispersion for RNA-seq data with DESeq2. *Genome Biol* **15**, 550 (2014).
11. McDowell, I.C. et al. Clustering gene expression time series data using an infinite Gaussian process mixture model. *PLoS Comput Biol* **14**, e1005896 (2018).
12. Sherman, B.T. et al. DAVID: a web server for functional enrichment analysis and functional annotation of gene lists (2021 update). *Nucleic Acids Res* **50**, W216-221 (2022).
13. Zhou, Y. et al. Metascape provides a biologist-oriented resource for the analysis of systems-level datasets. *Nat Commun* **10**, 1523 (2019).
14. Vento-Tormo, R. et al. Single-cell reconstruction of the early maternal-fetal interface in humans. *Nature* **563**, 347-353 (2018).
15. Karlsson, M. et al. A single-cell type transcriptomics map of human tissues. *Sci Adv* **7** (2021).
16. Saldanha, A.J. Java Treeview--extensible visualization of microarray data. *Bioinformatics* **20**, 3246-3248 (2004).

Supplementary Fig. 2d, CT29 time-course samples

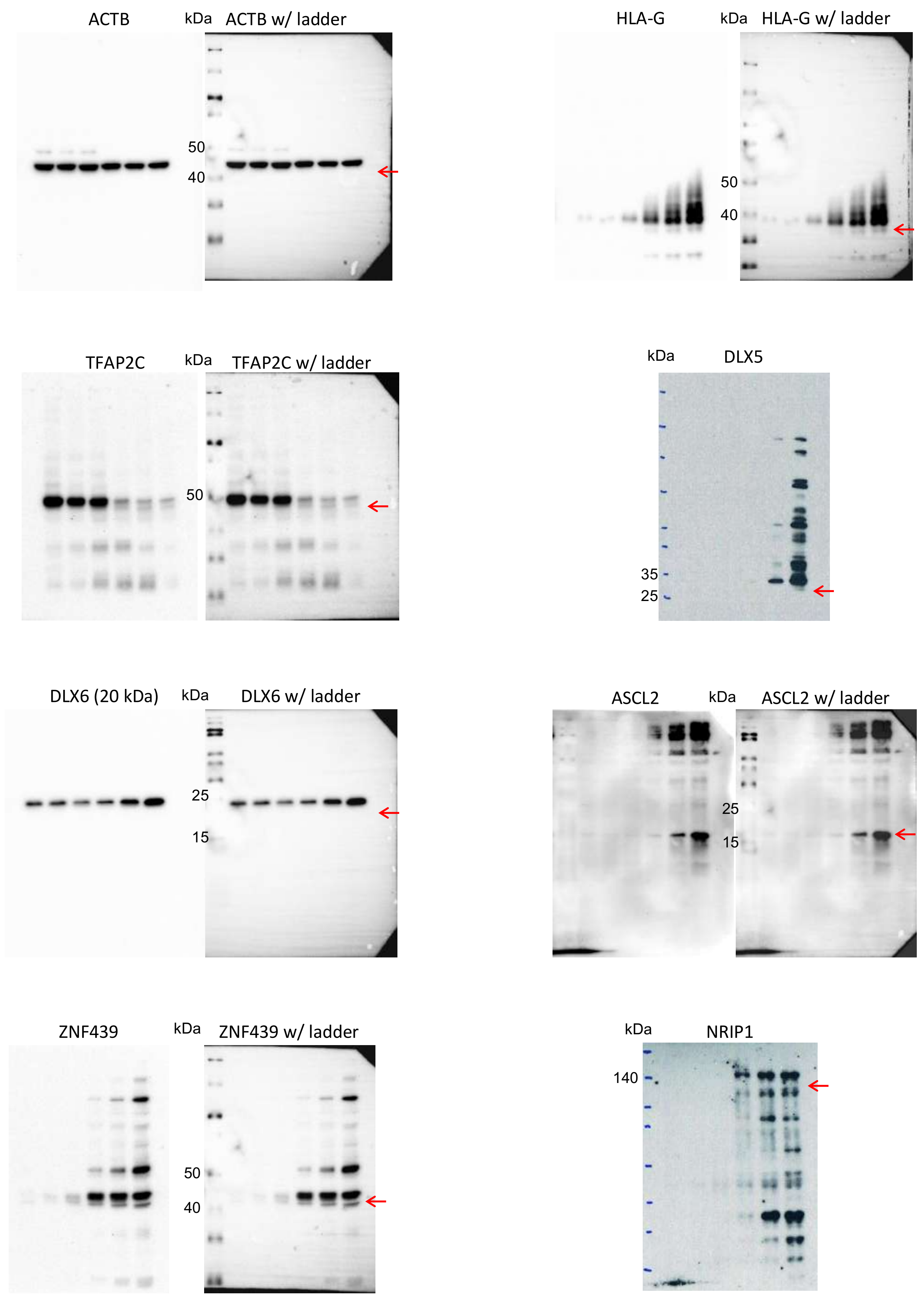

Supplementary Fig. 2d, CT30 time-course samples

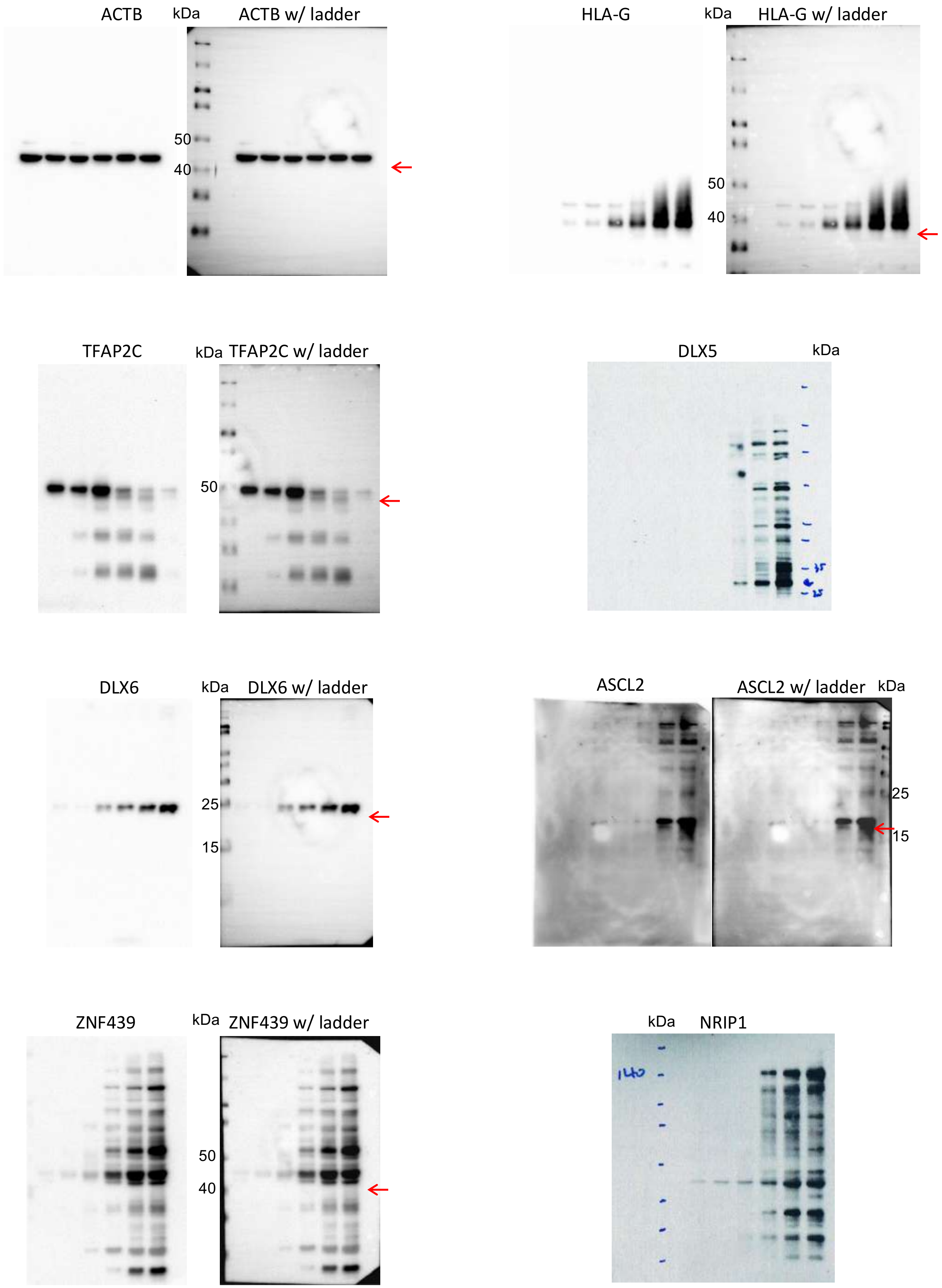

Supplementary Fig. 4g

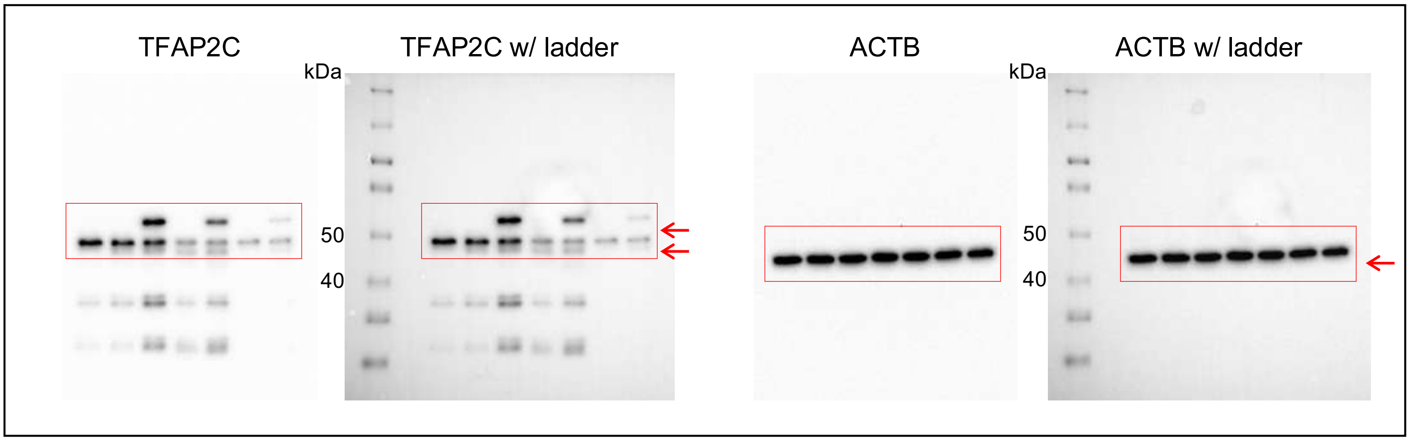

Supplementary Fig. 7f

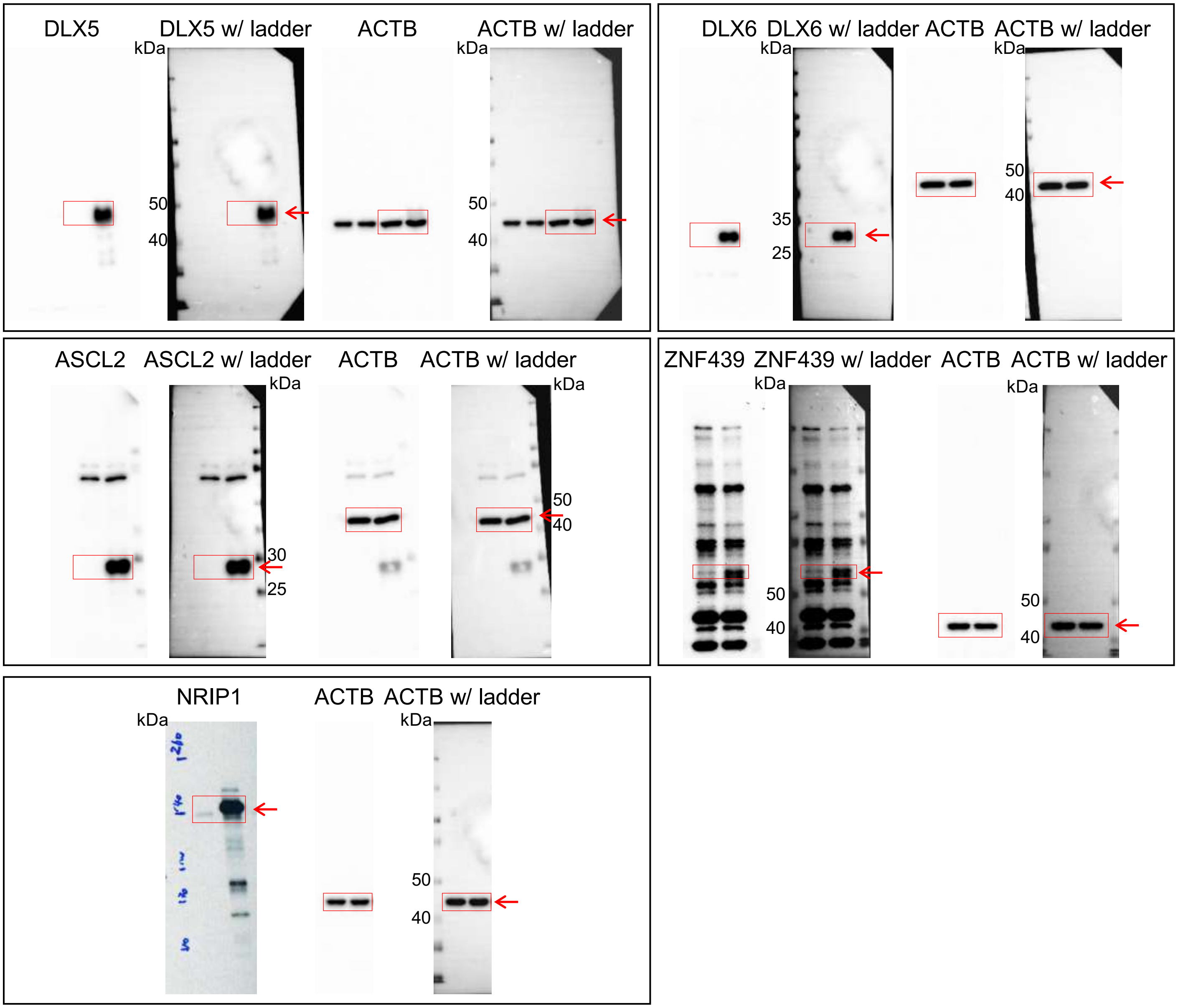

Supplement: Supplementary file 1 — Supplementary Information [file 41467_2024_45669_MOESM1_ESM.pdf]
