# Supplementary material for: The transcriptional regulatory network modulating human trophoblast stem cells to extravillous trophoblast differentiation
Source: Nat Commun. 2024 Feb 12;15:1285. doi: 10.1038/s41467-024-45669-2 (PMC10861538; doi:10.1038/s41467-024-45669-2)
Supplement: Supplementary file 3 — Description of Additional Supplementary Files [file 41467_2024_45669_MOESM3_ESM.pdf]

Description of Additional Supplementary Files

Supplementary Data 1. Count information of RNA-seq data

Supplementary Data 2. Class 1-4 genes and their changes in expression during EVT differentiation

Supplementary Data 3. Super-enhancer loci and associated genes

Supplementary Data 4. DEGs upon KD of EVT TFs

Supplementary Data 5. Genes associated with G1-G3 loci

Supplementary Data 6. Primers used for qPCR

Supplementary Data 7. Oligo sequences of shRNAs
