# Supplementary material for: The transcriptional regulatory network modulating human trophoblast stem cells to extravillous trophoblast differentiation
Source: Nat Commun. 2024 Feb 12;15:1285. doi: 10.1038/s41467-024-45669-2 (PMC10861538; doi:10.1038/s41467-024-45669-2)
Supplement: Supplementary file 11 — Reporting Summary [file 41467_2024_45669_MOESM11_ESM.pdf]

nature portfolio

Corresponding author(s): Jonghwan Kim

Last updated by author(s): Jan 18, 2024

Reporting Summary

Nature Portfolio wishes to improve the reproducibility of the work that we publish. This form provides structure for consistency and transparency in reporting. For further information on Nature Portfolio policies, see our Editorial Policies and the Editorial Policy Checklist.

Statistics

For all statistical analyses, confirm that the following items are present in the figure legend, table legend, main text, or Methods section.

n/a	Confirmed	
☐	☒	The exact sample size (*n*) for each experimental group/condition, given as a discrete number and unit of measurement
☐	☒	A statement on whether measurements were taken from distinct samples or whether the same sample was measured repeatedly
☐	☒	The statistical test(s) used AND whether they are one- or two-sided<br>*Only common tests should be described solely by name; describe more complex techniques in the Methods section.*
☒	☐	A description of all covariates tested
☒	☐	A description of any assumptions or corrections, such as tests of normality and adjustment for multiple comparisons
☐	☒	A full description of the statistical parameters including central tendency (e.g. means) or other basic estimates (e.g. regression coefficient) AND variation (e.g. standard deviation) or associated estimates of uncertainty (e.g. confidence intervals)
☐	☒	For null hypothesis testing, the test statistic (e.g. *F*, *t*, *r*) with confidence intervals, effect sizes, degrees of freedom and *P* value noted<br>*Give P values as exact values whenever suitable.*
☒	☐	For Bayesian analysis, information on the choice of priors and Markov chain Monte Carlo settings
☒	☐	For hierarchical and complex designs, identification of the appropriate level for tests and full reporting of outcomes
☒	☐	Estimates of effect sizes (e.g. Cohen's *d*, Pearson's *r*), indicating how they were calculated

Our web collection on statistics for biologists contains articles on many of the points above.

Software and code

Policy information about availability of computer code

Data collection	Salmon (v1.4.0, PMID: 28263959), Bowtie2 (v2.4.5, PMID: 22388286)
Data analysis	DESeq2 (v1.30.1, PMID: 25516281), Dirichlet Process Gaussian process (DPGP) mixture model (PMID: 29337990), tximport (PMID: 26925227), DAVID (PMID: 35325185), Metascape (PMID: 30944313), Bowtie2 (v2.4.5, PMID: 22388286), SAMtools (PMID: 19505943), deepTools (PMID: 27079975), IGV (PMID: 21221095), MACS3 (PMID: 18798982), HOMER (v4.11, PMID: 20513432), MAnorm (v1.3.0, PMID: 22424423), GREAT (PMID: 20436461), ROSE (PMID: 23582322, 23582323), Corrr (Kuhn, M., Jackson, S., & Cimentada, J. (2020). corrr: Correlations in R. R Package version 0.4, 2.) and pheatmap (v1.0.12, Kolde, R. (2019). pheatmap: Pretty Heatmaps. R package version 1.0. 12. CRAN. R-project. org/ package= pheatmap.)

For manuscripts utilizing custom algorithms or software that are central to the research but not yet described in published literature, software must be made available to editors and reviewers. We strongly encourage code deposition in a community repository (e.g. GitHub). See the Nature Portfolio guidelines for submitting code & software for further information.

Data

Policy information about availability of data

All manuscripts must include a data availability statement. This statement should provide the following information, where applicable:

- Accession codes, unique identifiers, or web links for publicly available datasets
- A description of any restrictions on data availability
- For clinical datasets or third party data, please ensure that the statement adheres to our policy

The sequencing data generated in this study have been deposited in the GEO database under accession code GSE212267.

Research involving human participants, their data, or biological material

Policy information about studies with human participants or human data. See also policy information about sex, gender (identity/presentation), and sexual orientation and race, ethnicity and racism.

Reporting on sex and gender	N/A
Reporting on race, ethnicity, or other socially relevant groupings	N/A
Population characteristics	N/A
Recruitment	N/A
Ethics oversight	N/A

Note that full information on the approval of the study protocol must also be provided in the manuscript.

Field-specific reporting

Please select the one below that is the best fit for your research. If you are not sure, read the appropriate sections before making your selection.

☒ Life sciences ☐ Behavioural & social sciences ☐ Ecological, evolutionary & environmental sciences

For a reference copy of the document with all sections, see nature.com/documents/nr-reporting-summary-flat.pdf

Life sciences study design

All studies must disclose on these points even when the disclosure is negative.

Sample size	Sample size calculation was not conducted for this study. ChIP-seq experiments involved one to three biological replicates for cell lines. RNA-seq experiments, except for time-course experiments (n=2, technical repeats), conducted a minimum of two biological replicates. For all other experiments (RT-qPCR, invasion assay, and flow cytometry), three biological replicates were used, except for Supplementary Fig. 2b and 4c (the 48 h sample), which had two biological replicates (n=2).
Data exclusions	Sequencing data for bioChIP-seq ofZNF439 and ASCL2 were excluded due to low data quality. Additionally, the bioChIP-seq data of NRIPl was not included in the analyses for Fig. 7c because it exhibited a unique occupancy pattern, as also described in the main text.
Replication	As described in the Sample Size section, ChIP-seq experiments involved one to three biological replicates for cell lines. RNA-seq experiments, except for time-course experiments (n=2, technical repeats), conducted a minimum of two biological replicates. Other experiments, including RT-qPCR, invasion assay, and flow cytometry, employed three biological replicates, except for Supplementary Fig. 2b and 4c (the 48 h sample), which had to biological replicates (n=2). All attempts at replication were successful.
Randomization	Randomization was not applicable to this manuscript since it was a cell-culture based study.
Blinding	Blinding was not applicable to this manuscript since it was a cell-culture based study.

Reporting for specific materials, systems and methods

We require information from authors about some types of materials, experimental systems and methods used in many studies. Here, indicate whether each material, system or method listed is relevant to your study. If you are not sure if a list item applies to your research, read the appropriate section before selecting a response.

Materials & experimental systems

n/a	Involved in the study
☐	☒ Antibodies
☐	☒ Eukaryotic cell lines
☒	☐ Palaeontology and archaeology
☒	☐ Animals and other organisms
☒	☐ Clinical data
☒	☐ Dual use research of concern
☒	☐ Plants

Methods

n/a	Involved in the study
☐	☒ ChIP-seq
☐	☒ Flow cytometry
☒	☐ MRI-based neuroimaging

Antibodies

Antibodies used

H3K27ac (Active Motif, 39133, Polyclonal)
H3K4me3 (Active Motif, 39159, Polyclonal)
DLX5 (Novus Biologicals, NBP1-85793, Polyclonal)
DLX6 (Proteintech, 23216-1-AP, Polyclonal)
DLX6 (abcam, ab137079, Monoclonal [EPR9474])
ASCL2 (Millipore, MAB4418, Monoclonal clone 7E2)
ASCL2 (R and D Systems, AF653, Polyclonal)
TFAP2C (Santa Cruz Biotechnology, sc-8977, Polyclonal)
TFAP2C (Santa Cruz Biotechnology, sc-12762, Monoclonal 6E4/4)
TFAP2C (Cell Signaling Technology, 2320, Polyclonal)
P300 (abcam, ab10485, Polyclonal)
MED1 (Bethyl Laboratories, A300-793A, Polyclonal)
H3K4me1 (abcam, ab8895, Polyclonal)
ZNF439 (Novus Biologicals, NBP2-13574, Polyclonal)
ZNF439 (GeneTex, GTX119735, Polyclonal)
NRIP1 (abcam, ab42126, Polyclonal)
NRIP1 (Millipore, MABS1917, Monoclonal clone 6D7)
HLA-G (Santa Cruz Biotechnology, sc-21799, Monoclonal 4H84)
MMP2 (Cell Signaling Technology, 40994, Monoclonal D4M2N)
TP63 (BioLogo, PP040-0.5, Monoclonal Zr8)
ACTB (Abgent, AM1829B, Monoclonal 137CT26.1.1)
phycoerythrin-conjugated HLA-G (abcam, ab24384, Monoclonal [MEM-G/9])

Validation

The original scan of Western blot results were included with the Source Data file or Supplementary information file (*). Additionally, validation from the manufacturer was provided as a website link for reference.

H3K27ac (Active Motif, 39133): https://www.activemotif.com/catalog/details/39133/histone-h3-acetyl-lys27-antibody-pab
H3K4me3 (Active Motif, 39159): https://www.activemotif.com/catalog/details/39159/histone-h3-trimethyl-lys4-antibody-pab
DLX5* (Novus Biologicals, NBP1-85793): https://www.novusbio.com/products/dlx5-antibody_nbp1-85793
DLX6 (Proteintech, 23216-1-AP): https://www.ptglab.com/products/DLX6-Antibody-23216-1-AP.htm
DLX6* (abcam, ab137079): https://www.abcam.com/products/primary-antibodies/dlx6-antibody-epr9474-ab137079.html
ASCL2 (Millipore, MAB4418): https://www.sigmaaldrich.com/US/en/product/mm/mab4418
ASCL2* (R and D Systems, AF653): https://www.rndsystems.com/products/human-ascl2-mash2-antibody_af6539
TFAP2C (Santa Cruz Biotechnology, sc-8977): https://www.scbt.com/p/ap-2gamma-antibody-h-77
TFAP2C* (Santa Cruz Biotechnology, sc-12762): https://www.scbt.com/p/ap-2gamma-antibody-6e4-4?requestFrom=search
TFAP2C (Cell Signaling Technology, 2320): https://www.cellsignal.com/products/primary-antibodies/ap-2g-antibody/2320
P300 (abcam, ab10485): https://www.abcam.com/products/primary-antibodies/kat3b--p300-antibody-ab10485.html
MED1 (Bethyl Laboratories, A300-793A): https://www.thermofisher.com/antibody/product/CRSP1-TRAP220-Antibody-Polyclonal/A300-793A
H3K4me1 (abcam, ab8895): https://www.abcam.com/products/primary-antibodies/histone-h3-mono-methyl-k4-antibody-chip-grade-ab8895.html
ZNF439 (Novus Biologicals, NBP2-13574): https://www.novusbio.com/products/znf439-antibody_nbp2-13574
ZNF439* (GeneTex, GTX119735): https://www.genetex.com/Product/Detail/ZNF439-antibody-N2C1-Internal/GTX119735
NRIP1 (abcam, ab42126): https://www.abcam.com/products/primary-antibodies/rip140-antibody-ab42126.html
NRIP1* (Millipore, MABS1917): https://www.emdmillipore.com/US/en/product/Anti-RIP140-Antibody-clone-6D7,MM_NF-MABS1917?ReferrerURL=https%3A%2F%2Fwww.google.com%2F
HLA-G* (Santa Cruz Biotechnology, sc-21799): Western blot result: https://www.scbt.com/p/hla-g-antibody-4h84
MMP2 (Cell Signaling Technology, 40994): Western blot result: https://www.cellsignal.com/products/primary-antibodies/mmp-2-d4m2n-rabbit-mab/40994
TP63 (BioLogo, PP040-0.5)
ACTB* (Abgent, AM1829B): https://www.abcepta.com/products/AM1829b-Beta-Actin-Antibody

Eukaryotic cell lines

Policy information about cell lines and Sex and Gender in Research

Cell line source(s)	Trophoblast stem cells (CT27, CT29, and CT30 lines) derived from human cytotrophoblasts (CTs) of the first-trimester human placenta (gift of Dr. Takahiro Arima, Tohoku University) were used. The CT lines used in this study were derived by Okae et al. (PMID: 29249463) from human placentas obtained from healthy women. The donors provided signed informed consent, and the study received approval from the Ethics Committee of Tohoku University School of Medicine (Research license 2014-1-879).<br>Detailed information about the cell lines can be found in RIKEN BRC CELL BANK website:<br>CT27 (https://cellbank.brc.riken.jp/cell_bank/CellInfo/?cellNo=RCB4936&lang=En)<br>CT29 (https://cellbank.brc.riken.jp/cell_bank/CellInfo/?cellNo=RCB4937&lang=En)<br>CT30 (https://cellbank.brc.riken.jp/cell_bank/CellInfo/?cellNo=RCB4938&lang=En)
Authentication	Following the knockdown or overexpression of the gene, RT-qPCR and Western blot analyses were performed.
Mycoplasma contamination	Cell lines were tested for mycoplasma and no mycoplasma contamination was detected.
Commonly misidentified lines<br>(See ICLAC register)	None of commonly misidentified cell lines were used.

Plants

Seed stocks	N/A
Novel plant genotypes	N/A
Authentication	N/A

ChIP-seq

Data deposition

- [x] Confirm that both raw and final processed data have been deposited in a public database such as GEO.
- [x] Confirm that you have deposited or provided access to graph files (e.g. BED files) for the called peaks.

Data access links<br>*May remain private before publication.*	GSE212265 (https://www.ncbi.nlm.nih.gov/geo/query/acc.cgi?acc=GSE212265)
Files in database submission	H3K27ac_TSC_comb.peak<br>H3K27ac_TSC_comb.peak<br>H3K27ac_EVT_D3_comb.peak<br>H3K27ac_EVT_D3_comb.peak<br>H3K27ac_EVT_D3_comb.peak<br>H3K27ac_EVT_D8_comb.peak<br>H3K27ac_EVT_D8_comb.peak<br>H3K27ac_EVT_D8_comb.peak<br>H3K4me3_TSC.peak<br>H3K4me3_EVT_D8.peak<br>ATAC_TSC.peak<br>ATAC_EVT_D8.peak<br>TFAP2C_EVT_D2.peak<br>TFAP2C_EVT_D5.peak<br>TFAP2C_EVT_D8.peak<br>DLX6_TSC.peak<br>DLX6_EVT_D2.peak<br>DLX6_EVT_D5.peak<br>DLX6_EVT_D8_comb.peak<br>DLX6_EVT_D8_comb.peak<br>ASCL2_EVT_D8_comb.peak<br>ASCL2_EVT_D8_comb.peak<br>DLX5_EVT_D8_comb.peak

DLX5_EVT_D8_comb.peak
NRIP1_EVT_D8.peak
TSC_H3K4me1_comb.final.peak.gz
TSC_Med1_comb.final.peak.gz
TSC_P300_comb.final.peak.gz
EVT3D_H3K4me1_comb.final.peak.gz
EVT3D_Med1_comb.final.peak.gz
EVT3D_P300_comb.final.peak.gz
EVT8D_H3K4me1_comb.final.peak.gz
EVT8D_Med1_comb.final.peak.gz
EVT8D_P300_comb.final.peak.gz

Genome browser session (e.g. UCSC)

N/A

Methodology

Replicates

H3K27ac for EVT day 3 and day 8 cells: n=3
Time-course ChIP-seq of TFAP2C and DLX6, H3K4me3, ATAC-seq: n=1 Others: n=2

Sequencing depth

H3K27ac_TSC_rep1: aligned read 57716303
H3K27ac_TSC_rep2: aligned read 61717533
H3K27ac_EVT_D3_rep1: aligned read 75775255
H3K27ac_EVT_D3_rep2: aligned read 59945616
H3K27ac_EVT_D3_rep3: aligned read 54430813
H3K27ac_EVT_D8_rep1: aligned read 60376294
H3K27ac_EVT_D8_rep2: aligned read 46473535
H3K27ac_EVT_D8_rep3: aligned read 62882123
H3K4me3_TSC: aligned read 64833443
H3K4me3_EVT_D8: aligned read 74227644
ATAC-seq_TSC: aligned read 67157556
ATAC-seq_EVT_D8: aligned read 45302181
TFAP2C_EVT_D2: aligned read 50832569
TFAP2C_EVT_D5: aligned read 48954421
TFAP2C_EVT_D8: aligned read 45677829
DLX6_TSC: aligned read 163566513
DLX6_EVT_D2: aligned read 51427188
DLX6_EVT_D5: aligned read 48954421
DLX6_EVT_D8_rep1: aligned read 46706716
DLX6_EVT_D8_rep2: aligned read 41345294
ASCL2_EVT_D8_rep1: aligned read 63039061
ASCL2_EVT_D8_rep2: aligned read 42567615
DLX5_EVT_D8_rep1: aligned read 44889936
DLX5_EVT_D8_rep2: aligned read 52577336
bioNRIP1_EVT_D8: aligned read 52254146
Input_TSC: aligned read 34393562
Input_EVT_D3: aligned read 38833055
Input_EVT_D8: aligned read 34817913
P300_TSC_Rep1: aligned read 44988831
P300_TSC_Rep2: aligned read 53782315
P300_EVT_D3_Rep1: aligned read 45030671
P300_EVT_D3_Rep2: aligned read 51894687
P300_EVT_D8_Rep1: aligned read 39424284
P300_EVT_D8_Rep2: aligned read 37866519
MED1_TSC_Rep1: aligned read 53027935
MED1_TSC_Rep2: aligned read 44702518
MED1_EVT_D3_Rep1: aligned read 52996814
MED1_EVT_D3_Rep2: aligned read 49933137
MED1_EVT_D8_Rep1: aligned read 39770182
MED1_EVT_D8_Rep2: aligned read 30691335
H3K4me1_TSC_Rep1: aligned read 69980340
H3K4me1_TSC_Rep2: aligned read 59141976
H3K4me1_EVT_D3_Rep1: aligned read 73717611
H3K4me1_EVT_D3_Rep2: aligned read 66779795
H3K4me1_EVT_D8_Rep1: aligned read 70980377
H3K4me1_EVT_D8_Rep2: aligned read 68507061

Antibodies

H3K27ac (Active Motif, 39133, 10 μg)
H3K4me3 (Active Motif, 39159, 10 μg)

	DLX5 (Novus Biologicals, NBP1-85793, 20 μL)<br>DLX6 (Proteintech, 23216-1-AP, 20 μL)<br>ASCL2 (Millipore, MAB4418, 20 μL)<br>TFAP2C (Santa Cruz Biotechnology, sc-8977, 10 μg)<br>P300 (abcam, ab10485, 4 μL)<br>MED1 (Bethyl Laboratories, A300-793A, 5 μL)<br>H3K4me1 (abcam, ab8895, 4 μL)
Peak calling parameters	macs3 callpeak -t $path/Sample.bam -c $path/Input.bam -f BAM -g hs -B -q 0.01 --outdir $path/Sample
Data quality	Significant correlation was observed among replicates, and there was enrichment of peaks to cis-regulatory elements of marker genes. Additionally, the binding patterns to time-course RNA-seq data were as expected.
Software	Bowtie2 (v2.4.5, PMID: 22388286), SAMtools (PMID: 19505943), deepTools (PMID: 27079975), IGV (PMID: 21221095), MACS3 (PMID: 18798982), HOMER (v4.11, PMID: 20513432), MAnorm (v1.3.0, PMID: 22424423), GREAT (PMID: 20436461), ROSE (PMID: 23582322, 23582323), Corrr (Kuhn, M., Jackson, S., & Cimentada, J. (2020). corrr: Correlations in R. R Package version 0.4, 2.) and pheatmap (v1.0.12, Kolde, R. (2019). pheatmap: Pretty Heatmaps. R package version 1.0. 12. CRAN. R-project. org/package= pheatmap.)

Flow Cytometry

Plots

Confirm that:

- [x] The axis labels state the marker and fluorochrome used (e.g. CD4-FITC).
- [x] The axis scales are clearly visible. Include numbers along axes only for bottom left plot of group (a 'group' is an analysis of identical markers).
- [x] All plots are contour plots with outliers or pseudocolor plots.
- [x] A numerical value for number of cells or percentage (with statistics) is provided.

Methodology

Sample preparation	Cells were dissociated into single cells using TrypLE and then washed with phosphate-buffered saline. The cells were subsequently resuspended in 200 μL of 1x Annexin V binding buffer with 5 μL of Sulforhodamine 101-Annexin V (Texas Red) stock solution and incubated for 30 minutes at room temperature, while being protected from light. Afterward, the cells were washed once with 1x Annexin V binding buffer and filtered through a 70 μm cell strainer (Falcon, 352235).
Instrument	BD LSRFortessa SORP Flow Cytometer (BD Biosciences)
Software	FlowJo (v9, Treestar)
Cell population abundance	The quantification of HLA-G positively stained cells was performed using FlowJo software, as shown in Supplementary Fig. 1b. Additionally, Annexin V signals (conjugated to CF594) and caspase-3 activity (NucView 488) were measured using FlowJo software, as depicted in Supplementary Fig. 4d. For detailed information about the cell numbers, please refer to the manuscript.
Gating strategy	Cell debris were excluded using FSC-A vs. SSC-A gate, while aggregates were excluded via FSC-A vs. FSC-H.

- [x] Tick this box to confirm that a figure exemplifying the gating strategy is provided in the Supplementary Information.
